# Heart Sound Classification Network Based on Convolution and Transformer

**DOI:** 10.3390/s23198168

**Published:** 2023-09-29

**Authors:** Jiawen Cheng, Kexue Sun

**Affiliations:** 1 College of Electronic and Optical Engineering & College of Flexible Electronics (Future Technology), Nanjing University of Posts and Telecommunications, Nanjing 210023, China; 1021020904@njupt.edu.cn; 2 Nation-Local Joint Project Engineering Laboratory of RF Integration & Micropackage, Nanjing 210023, China

**Keywords:** electronic auscultation, CVDs, heart sound classification, neural network, one-dimensional convolution, Transformer encoder

## Abstract

Electronic auscultation is vital for doctors to detect symptoms and signs of cardiovascular diseases (CVDs), significantly impacting human health. Although progress has been made in heart sound classification, most existing methods require precise segmentation and feature extraction of heart sound signals before classification. To address this, we introduce an innovative approach for heart sound classification. Our method, named Convolution and Transformer Encoder Neural Network (CTENN), simplifies preprocessing, automatically extracting features using a combination of a one-dimensional convolution (1D-Conv) module and a Transformer encoder. Experimental results showcase the superiority of our proposed method in both binary and multi-class tasks, achieving remarkable accuracies of 96.4%, 99.7%, and 95.7% across three distinct datasets compared with that of similar approaches. This advancement holds promise for enhancing CVD diagnosis and treatment.

## 1. Introduction

CVD is one of the most common causes of disability and death worldwide, seriously impacting human health and quality of life. It is expected that from 2025 to 2060, the percentage of CVD cases in the US, particularly stroke and heart failure, will sharply increase. Moreover, global reports indicate that there was an increase in excess deaths caused by cardiovascular disease during the COVID-19 pandemic. This increase suggests limited access to preventive CVD services during the pandemic and reduced monitoring of cardiovascular risk factors and behaviors [1]. However, with the help of auxiliary diagnosis, the efficiency and accuracy of diagnosis can be improved, and remote diagnosis and treatment can also be achieved. This can expand the opportunities for obtaining preventive CVD services without increasing the existing medical resources.

**Figure 1:**
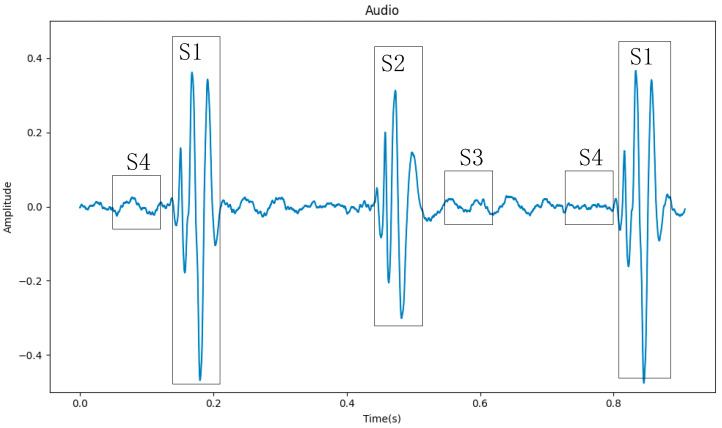
A normal heart sound phonocardiogram (PCG).

Heart sound is one of the body’s natural signals and the external reflection of the human heart’s physiological information [2]. Doctors make a preliminary determination of the patient’s heart disease by auscultation. And, some CVDs can be diagnosed through auscultation, such as murmurs, extra heart (extrah). Using an electronic stethoscope, heart sounds can be converted into phonocardiograms (PCGs), which can be processed on a computer to aid doctors in diagnosis, as shown in Figure 1. Heart sound signals are mainly composed of four segments (S1, S2, S3, and S4), of which S1 and S2 contain the main information of the heart sound signal [3]. Because the S3 segment is low-pitched, weak in intensity, and short in duration, it is usually only heard in children and teenagers. The S4 segment appears at the end of ventricular diastole, and if it can be heard, it is mostly pathological. So, in Figure 1, the S3 and S4 segments do not stand out.

**Figure 2:**
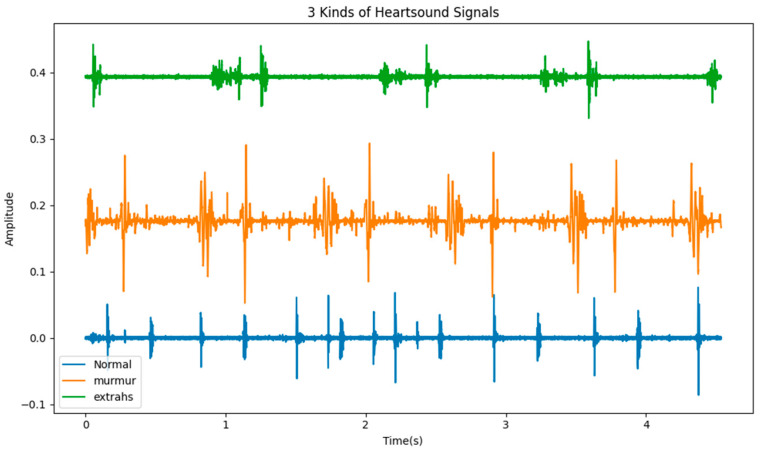
Three kinds of heart sound signals.

The PCGs of different types of heart sounds also have obvious differences, as shown in Figure 2. We can easily tell the difference between concentrated heart sound signals. Therefore, the automatic detection of heart abnormalities through heart sound signals has garnered the interest of numerous researchers. This topic can be regarded as a multidisciplinary research area that also encompasses telemedicine. In recent years, researchers have carried out a lot of work to improve the accuracy and efficiency of heart sound signal classification.

Early heart sound signal classification research mainly focused on traditional signal processing techniques and machine learning algorithms. These methods typically required manual feature extraction and classifier design, lacked generalization ability, and had lower accuracy. However, these methods provided the foundation for later deep-learning approaches.

Maglogiannis et al. [4] segmented the heart sound signals into S1 and S2 segments, then employed morphological transformation for feature extraction, and utilized a support vector machine (SVM) for automated classification. This system achieved an accuracy of 91.43%. In [5], a dynamic feature for classification was discussed. They calculated the time–frequency spectrum power of heart sound to detect systolic murmurs. The method showed excellent performance on the established dataset, with an accuracy of up to 98%. Pantea et al. [6] extracted MFCC from the heartbeat sounds. Then, the proposed Adaptive Neuro-Fuzzy Inferences System using an artificial bee colony was used to run the pre-processed features and achieved 93% accuracy for the murmur class.

The development of deep learning [7] can be traced back to the 1980s. Deep learning methods can automatically extract features and have strong generalization ability. It has achieved great success in fields such as image detection and speech recognition. In recent years, many scholars have introduced deep learning methods into heart sound signal classification, greatly improving the accuracy and efficiency of heart sound signal classification.

Most scholars commonly adopt the approach of first extracting features from heart sound signals and then feeding these features into a constructed network for modeling and classification. Nilanon et al. [8] extracted the power spectral density (PSD) and Mel-frequency cepstral coefficients (MFCCs) as pre-extracted features. Then, they used a convolutional neural network (CNN) to capture local changes of pre-extracted features. And, this method obtained a score of 81.3% on the dataset of the 2016 PhysioNet/CinC Challenge [9]. Chen et al. [10] combined Mel-frequency spectrum and Log-Mel-frequency spectrum features of heart sound signals to improve performance based on CNN. In [11], the heart sound signals were converted to a Log-Mel-frequency spectrum. And, they used a Transformer-based architecture for classification.

Some scholars first segmented the S1 and S2 segments of the heart sound signals and then proceeded with operations such as feature extraction. This approach helps avoid the influence of other cardiac cycles on the heart sounds. Rubin et al. [12] used the Springer segmentation algorithm to segment each heart sound signal waveform into basic heart sounds (S1, S2, S3, and S4) and then selected a 3 s heart sound segment starting from the S1 segment, extracted MFCC as the input of the CNN, and achieved a score of 84.8%. The paper in [13] used an improved duration-dependent hidden Markov model (DHMM) to segment the heart sound signals based on the cardiac cycle. Then, they extracted Log-Mel-frequency spectral coefficient (MFSC) features using the dynamic frame length method as inputs of CNN. The article achieved a binary classification accuracy of 93.89% and a multi-class classification accuracy of 86.25% on the established dataset.

A smaller number of researchers directly inputted heart sound signals into a network for feature extraction and classification after performing some simple preprocessing. Li et al. [14] normalized, filtered, and segmented the heart sound signals. These processed signals were then used as input for a Heart Sound Feature Extraction network based on CNN and Group Convolution, achieving an accuracy of 95.5%. Similarly, Xiao et al. [15] conducted basic preprocessing on the signals before feeding them into a lightweight network based on Clique-CNN with minimal parameters. This approach resulted in an accuracy of 93.28%.

Until now, many experts have made great progress in the field of heart sound signal processing. However, there is still a lot of room for improvement in the following areas: heart sound signal denoising, segmentation, and feature extraction, which all require complex algorithms to implement. In addition, some methods segment the S1 and S2 intervals of the heart sound signal for classification. But, this method requires precise segmentation to achieve accurate classification; otherwise, it will greatly affect the subsequent classification accuracy.

In conclusion, directly processing heart sound signals and inputting them into a neural network for classification is a feasible and efficient approach. Firstly, by directly feeding the raw signals into the neural network for training, it enables an end-to-end learning process, reducing intermediate processing steps. This helps prevent information loss and premature feature extraction. Secondly, feature pre-extraction and precise segmentation may require multiple steps and parameter adjustments, increasing the complexity of the processing flow. Using an end-to-end learning approach directly can reduce these processing steps and streamline the process. Lastly, heart sound signals may contain intricate temporal and frequency patterns that might not be easily captured with traditional feature extraction methods. Neural networks possess strong non-linear modeling capabilities, allowing them to better capture these complex patterns.

Therefore, this paper proposes a method for heart sounds classification using a neural network composed of a one-dimensional convolutional module and a Transformer encoder for feature extraction. The normalized heart sound sequence is directly used as the input to the network, without performing feature pre-extraction or segmentation on the heart sound signal. After training, the network outputs the classification results.

Compared with other classification methods such as Decision Tree (DT), Random Forest (RF), Linear Support Vector Machine (LSVM), Multi-Layer Perceptron Neural Network (MLP), and Recurrent Neural Network (RNN), the approach proposed in this paper has distinct advantages. We utilize a CNN to capture local patterns and features, and a Transformer encoder to model long-range dependencies within sequences, thereby comprehensively understanding information within the signal across multiple levels.

This paper is organized as follows: Section 2 covers the methodology; Section 3 presents the experimental, results, and analysis; Section 4 discusses the results. Section 5 presents the conclusions and prospects.

## 2. Methodology

### 2.1. One-Dimensional Convolution

Convolution is a commonly used operation in deep learning, especially in CNN for tasks such as image recognition, speech recognition, and natural language processing. The convolution operation can be thought of as a special type of weighted averaging operation, which mainly works by convolving input data with a set of learnable convolutional kernels. The convolutional layer typically consists of multiple convolutional kernels, which can extract different feature information from the data. The convolution process can be expressed using Equation (1).

(1) $$y_{i}\left(t\right)=x\left(t\right)*h_{i}\left(k\right)$$

where $x\left(t\right)$ represents the input data, $h_{i}\left(k\right)$ represents the weight values of the convolutional kernel of size *k*, ∗ represents a dot product operation, and $y_{i}\left(t\right)$ represents the output data obtained by convolving the input data with the convolutional kernel.

Two-dimensional and three-dimensional convolutions are usually applied in fields such as image processing, video processing, computer vision, etc. While one-dimensional convolution is commonly used in areas such as speech recognition, natural language processing, etc. This is because one-dimensional convolution is an operation that performs convolution on a single dimension (usually the time dimension). It perceives the input signal locally through a sliding window and extracts local features of the input signal. The extracted features may include information such as frequency, amplitude, and slope of the signal. By the above steps, 1D-conv achieves modeling and analysis of sequential data.

**Figure 3:**
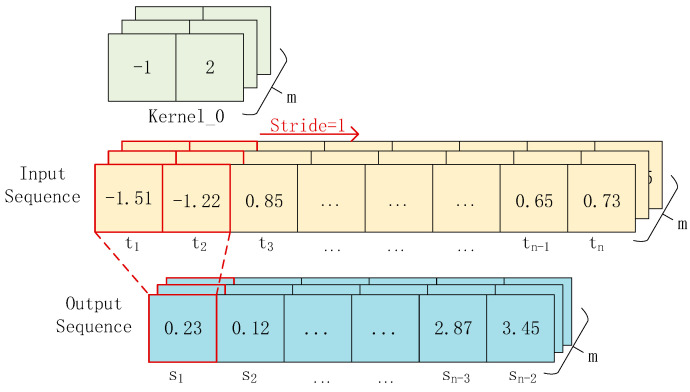
Principle of one-dimensional convolution.

One-dimensional convolution does not mean that the convolutional kernel has only one dimension or that the feature being convolved is one-dimensional. The “one-dimensional” refers to the direction in which the convolution is performed, which is usually along the time axis. The input of one-dimensional convolution is generally a three-dimensional matrix of shape [*batch_size*, *n*, *m*], where the *batch*_*size* refers to the number of feature maps input at once, *n* refers to the length of the feature map along the time axis, and *m* refers to the feature dimension within a single time unit. Figure 3 illustrates the principle of one-dimensional convolution. The input is a [*n* × *m*] sequence. After convolving it with a convolutional kernel of size 2, we obtain an output sequence of size [*n* × *m*]. When there are *k* such convolutional kernels, we can obtain *k* feature sequences.

### 2.2. Transformer Encoder

Because speech signals, heart sounds, and other signals are a type of time-series sequence, recurrent neural networks (RNNs) [16] and long short-term memory networks (LSTMs) [17] can naturally handle temporal information when processing such signals. They can transmit and remember previous states in the network, enabling them to capture temporal features of the signal, such as its temporal structure, duration, and changes.

However, despite the advantages of RNNs and LSTMs in speech processing and recognition, there are also some limitations. In traditional RNNs, each time step performs operations on the current input and the hidden state from the previous time step to obtain the current hidden state. Since the gradient is multiplied at each time step, if the gradient at each time step is less than 1, the gradient value will approach 0 after multiple time steps of multiplication, leading to the vanishing gradient problem. This can cause RNNs to be unable to remember information over long time intervals. LSTMs solves the vanishing gradient problem by introducing gate mechanisms. However, when the sequence length is very long, LSTMs also faces the problems of vanishing gradients and exploding gradients. Additionally, since LSTMs needs to maintain two state variables, as the number of time steps increases, these state variables will become larger and lead to the problem of exploding gradients.

Therefore, to solve these problems, Vaswani et al. [18] from Google Brain proposed the Transformer model in 2017. Transformer is a neural network architecture for sequence modeling based on the self-attention mechanism, which considers all positions of the input sequence for computation. It not only enables parallel computation, greatly reducing training time, but also captures global dependencies in the input sequence very well.

**Figure 4:**
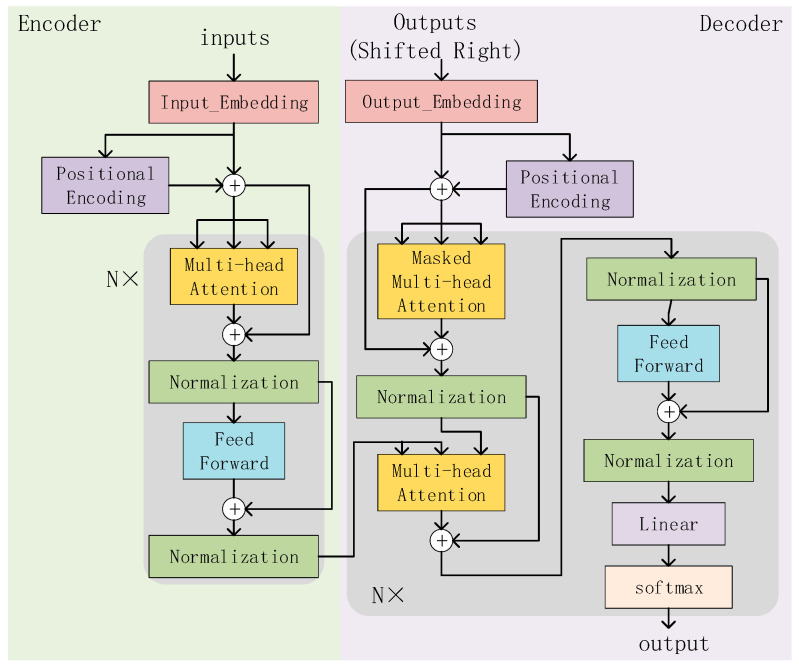
Structure of Transformer.

The overall structure of the Transformer is shown in Figure 4, where N = 6, indicating that it consists of six stacked structures. To reduce computational complexity and simplify the network structure, we only use the Transformer’s encoder module for feature extraction. The encoder can model each position in the sequence and capture relationships between different positions. Finally, the output of the encoder layer is used as the input of the classifier to complete the heart sound classification task. The encoder module mainly consists of three steps:

(i) Positional encoding

The self-attention mechanism only considers the mutual relationships between words in the input sequence, but not their positional relationships in the input sequence. So positional encoding is needed to represent the position information of words in the input sequence. The formula for positional encoding is given by Equations (2) and (3).

(2) PE2i=sin⁡pos10002idmodel

(3) PE2i+1=cos⁡pos10002idmodel

where i represents the position being computed and $d_{model}$ represents the dimensionality of the feature at each position, which is determined by the input. ${PE}_{2i}$ represents the encoding value for even positions, and ${PE}_{2i+1}$ represents the encoding value for odd positions. A matrix is obtained with the same size as the input feature map, and this matrix is added to the feature map to complete the position encoding.

(ii) Multi-Head Attention

Multi-Head Attention is one of the core components of the Transformer. It is a method of performing self-attention in different representation subspaces. And, it allows the model to focus on different parts of the input while maintaining computational efficiency.

**Figure 5:**
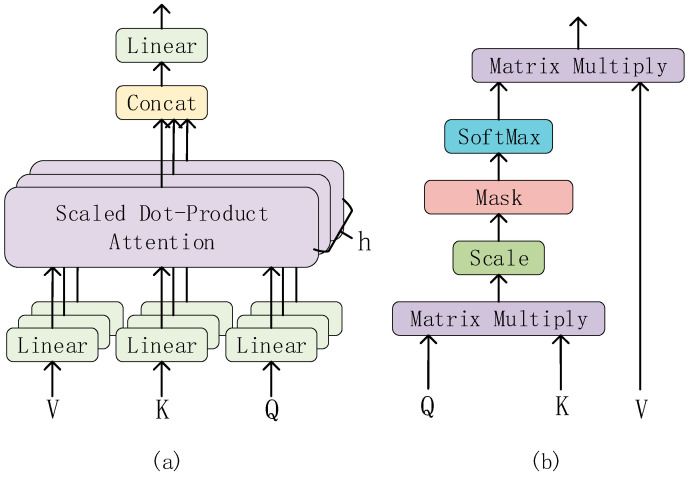
(**a**) Structure of the Multi-Head Attention; (**b**) Scaled Dot-Product Attention.

The structure of the Multi-Head Attention is shown in Figure 5a. The input of the Multi-Head Attention consists of three vectors: the Query vector (Q), the Key vector (K), and the Value vector (V). In each Multi-Head Attention sublayer, these three vectors are linearly transformed into h groups (heads) of different feature vectors, which are then input into the Scaled Dot-Product Attention (structure as shown in Figure 5b).

In the Scaled Dot-Product Attention layer, the two matrices Q and K are multiplied first to obtain the original attention score matrix (attention weight matrix). Then, in order to avoid the gradient problem caused by excessive scores, the attention score matrix is scaled (Scale layer) and the scores are equalized. Then, it is passed through the mask layer, which is a mask matrix that, when multiplied with the scaled attention score matrix, sets the unwanted part to infinity. Then, through the softmax layer, the attention score is normalized. Because the unnecessary part is set to infinity, after passing the softmax layer, the unnecessary part will infinitely tend to 0. So, the attention weight is obtained. Finally, the weighted value matrix is obtained by multiplying the normalized attention weight matrix with the value matrix V.

Then, these feature vectors are concatenated, and after another linear transformation, the final Multi-Head Attention output is obtained.

### 2.3. Fully Connected Module

The fully connected layer has different roles at different positions. In the middle layers, the fully connected layer can map high-dimensional features to low-dimensional ones. It achieves the function of dimensionality reduction and feature compression. In the final output layer, the fully connected layer, in conjunction with the softmax activation function, can map the features of the last layer to the probability distribution of the categories to achieve the classification task.

**Figure 6:**
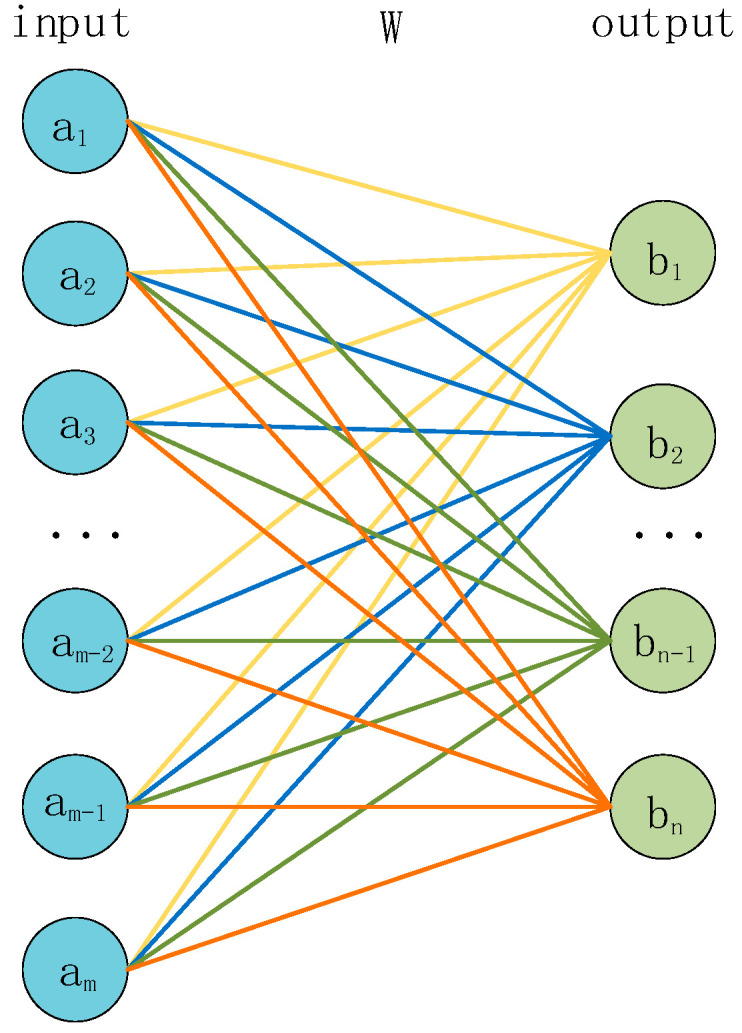
Structure of the fully connected layer.

The structure of the fully connected layer is shown in Figure 6. The input *a* is multiplied by a weight matrix *W* to obtain the corresponding output *b*, and the size of the weight matrix W ([*m* × *n*]) is determined by the size of the input and output. The formula for the fully connected layer is shown in Equation (4).

(4) $$y=softmax\left(Wx+b\right)$$

where x is the input vector; W is the weight matrix; y is the output vector; and b is the bias which is used to offset the results of the linear transformation, which makes the final output more flexible and adaptive to different tasks and data. softmax is the activation function, and its formula is shown in Equation (5).

(5) $${\sigma (x)}_{j}=\frac{e^{x_{j}}}{\sum_{k=1}^{n}e^{x_{k}}}$$

where j=1,2,…,n, x is an n-dimensional input vector and σ(x) is an output vector representing the probability distribution after transformation by the softmax function.

For a classification problem with n classes, the neural network will output *n* numbers, representing the probability scores of belonging to each class. These probability scores do not necessarily satisfy the properties of a probability distribution, which means they may not be non-negative and their sum may not be 1. The softmax function transforms these n numbers into a probability distribution, making them non-negative and summing up to 1.

### 2.4. Preprocessing of Heart Sound Signals

Preprocessing of heart sound signals is performed to remove noise, improve signal quality, and normalize the input for subsequent signal processing and analysis. Here are some common methods for preprocessing heart sound signals.

(i) Denoising

Heart sound signals are important physiological signals that have significant implications for the diagnosis and treatment of cardiovascular diseases. However, during the process of acquisition and transmission, heart sound signals are often subject to various types of interference and noise, such as respiratory noise, motion noise, environmental noise, etc. These noises can affect the accuracy and reliability of the signals, making the diagnosis and treatment of cardiovascular diseases difficult.

Therefore, before classifying heart sound signals, it is necessary to perform denoising processing on the signals. Denoising is an important step in signal processing, which aims to remove the noise component from the signal to improve the signal-to-noise ratio and accuracy. The frequency of heart sound is mainly concentrated in 20–150 Hz; to obtain more information of heart sound signal, this frequency band signal needs to be highlighted [19]. In this study, a fourth-order Butterworth bandpass digital filter with a passband of 25 Hz to 400 Hz was used for filtering.

(ii) Normalization

Normalization refers to scaling the amplitude range of a signal to a fixed range so that different signals can be compared and processed. Normalization is very common in signal processing, especially in deep learning. Due to the presence of activation functions, the amplitude range of the signal has an important impact on the training and performance of neural networks.

Specifically, in heart sound signal classification, normalization can help to solve the following two problems:

(a) Eliminating the amplitude difference between signals from different devices or environments: Since heart sound signals collected from different devices or environments have different amplitude ranges, this can affect the processing and classification of the signals. Therefore, normalization of the signals is required to eliminate this amplitude difference so that the signals can be compared and processed.

(b) Improving the stability and training effectiveness of neural networks: In neural networks, the role of the activation function is to map the input signal to a certain range. If the amplitude range of the input signal is too large, it will cause problems such as activation function saturation and gradient disappearance, which will affect the training effectiveness and stability of the neural network. Therefore, normalization of heart sound signals can help to scale the amplitude range of the signals to an appropriate range, thereby improving the training effectiveness and stability of the neural network. The normalization formula is shown in Equation (6).

(6) $$y_{i}=\frac{x_{i}}{\max(|x|)}$$

where x is an n-dimensional input, i=1,2,…,n, max⁡(|x|) refers to the maximum absolute value among the numbers in the input, and y is the output.

(iii) Downsampling

Downsampling refers to reducing the sampling rate of a signal to a lower frequency. Since the original heart sound signal usually has a high sampling rate and large time duration, it is computationally expensive and increases the complexity of the model, leading to slow training and overfitting issues. Therefore, downsampling can effectively reduce the time scale of the signal, making it easier to handle, while also reducing the computation and improving training efficiency and generalization ability.

(iv) Segmentation

Different from dividing heart sounds into basic heart sounds mentioned earlier, segmentation here refers to directly cutting the heart sound signal into fixed-length segments without detection. It can facilitate subsequent feature extraction and model training.

The heart sound signal is usually a very long time series signal containing many complex cardiac events and physiological information, which may be distributed in different positions throughout the signal. If the entire signal is classified directly, it is difficult to make full use of this information, while also increasing the complexity and computational resources of the model. Therefore, dividing the signal into fixed-length segments can effectively improve the processing efficiency of the signal and the generalization ability of the model, as well as increase the number of datasets.

A heart sound signal cycle is approximately 0.8 s, but due to individual differences, it is set to 2.5 s, which ensures at least 1–2 heart cycle periods. In addition, to increase the amount of data, the overlap of the sliding window is set to 50%.

## 3. Experiment and Results

**Figure 7:**
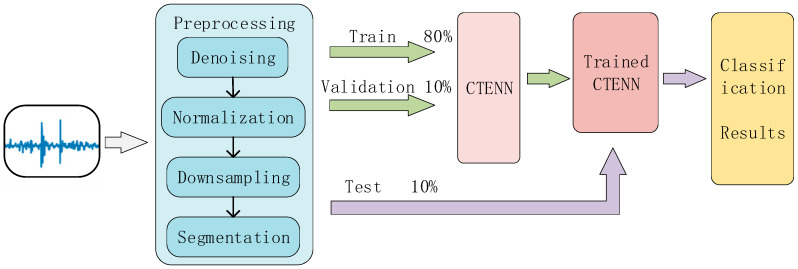
Process of the heart sound signal classification.

The specific process of the heart sound signal classification method proposed in this paper is shown in Figure 7.

First, we preprocess the data in each dataset to normalize signals of different lengths, sampling rates, and sampling environments. Each wav file in each dataset is denoised using a fourth-order Butterworth filter, and then, the signal intensity is normalized. In order to reduce the reduction of parameters and training time, the signal is downsampled to 2000 Hz. Finally, each wav file is divided into several fragments according to 2.5 s, and the fragments of each dataset constitute a dataset.

The 90% fragments of each dataset were sent as training sets to the constructed neural network for feature extraction and classification of heart sounds; 80% of this is for training and 10% is for validation.

Finally, the remaining 10% fragments are sent into the trained neural network as a test set for testing and evaluation, and the performance data of the network are obtained.

### 3.1. Datasets

During the process of collecting heart sound signals, it is easily affected by noise interference. Therefore, we have selected open-source datasets that are commonly used. Datasets *a* and *b* are the original datasets, while dataset *c* is a new dataset we created by combining several original datasets.

*a*. The 2016 PhysioNet/CinC Challenge dataset includes heart sound recordings from multiple participants from around the world, including healthy subjects and pathological patients in clinical or non-clinical environments. The challenge training set consists of five databases (a to e), totaling 3126 heart sound recordings, with durations ranging from 5 s to 120 s, and containing two labels: normal and abnormal. All recordings have been resampled to 2000 Hz and provided in .wav format.

*b*. Son et al. [20] collected a dataset that includes 1000 normal and abnormal heart sound files, with five labels: normal, AS, MS, MR, and MVP. All recordings have been resampled to 8000 Hz and provided in .wav format.

*c*. Based on datasets *a* and *b*, we also added two datasets collected by Peter Bentley et al. [21] for the PASCAL Heart Sound Classification Challenge. The dataset contains two sub-datasets, with dataset A including four labels: normal, murmur, extra heart sound, and artifact. Dataset B includes three labels: normal, murmur, and extrasystole. We grouped all non-normal heart sound signals into the abnormal category, and normal heart sounds into the normal category.

After preprocessing, the datasets *a*, *b*, and *c* contain the number of samples, as shown in Table 1. Then, we split each dataset for experimentation in a ratio of train/validation/test (8:1:1).

### 3.2. Evaluation

(1) Confusion Matrix

The confusion matrix, also known as an error matrix, is a standard format for representing accuracy evaluation. The horizontal axis represents the predicted label value, and the vertical axis represents the true label value. The numbers in the matrix represent the number of data points where the true label a was predicted as label b. Here, a and b can be any labels on the vertical and horizontal axis for binary classification models. True negative (TN) refers to the number of times 0 is predicted as 0. False negative (FN) refers to the number of times 0 is predicted as 1. False positive (FP) refers to the number of times 1 is predicted as 0. True positive (TP) refers to the number of times 1 is predicted as 1.

(2) Sensitivity (Se)

Sensitivity, also known as recall or true positive rate (TPR), refers to the proportion of true positive samples among all positive samples in the true labels. The calculation formula is shown in Equation (7).

(7) Se=recall=TPR=TP/(TP+FN)

(3) Specificity (Sp)

Specificity, also known as false positive rate (FPR), refers to the proportion of true negative samples among all negative samples in the true labels. The calculation formula is shown in Equation (8).

(8) Sp=1−FPR=TN/(TN+FP)

(4) Accuracy

Accuracy refers to the proportion of all correctly predicted samples among all tested samples. The calculation formula is shown in Equation (9).

(9) Acc=(TP+TN)/(TP+TN+FP+FN)

(5) Receiver Operating Characteristic (ROC)

The ROC curve is a graphical tool used to assess the performance of classification models. It illustrates the relationship between the true positive rate (TPR), also known as sensitivity, and the false positive rate (FPR) of the model at different classification thresholds. The area under the curve (AUC-ROC) is commonly used to measure the overall performance of the model. The value of AUC-ROC ranges between 0 and 1, with values closer to 1 indicating better model performance.

Its drawing method and calculation method are as follows:

(i) Use a classification model to predict the test data, and obtain the predicted probability or direct predicted category of the model for each sample.
(ii) According to the predicted probability or category, calculate a series of corresponding FPR and TPR according to different thresholds (from 0 to 1).
(iii) Plot FPR on the horizontal axis and TPR on the vertical axis to form an ROC curve. The closer the curve is to the upper left corner, the better the model performance.
(iv) Calculate the area under the curve (AUC-ROC), which measures the overall performance of the model. The larger the AUC value, the better the model performance.

### 3.3. Experimental Setting

The method proposed in this paper was implemented on an NVIDIA 3090Ti graphics card. The proposed neural network was implemented using Keras and the sparse categorical cross-entropy loss function was used as the loss function to save memory and to achieve higher classification accuracy. The Adam optimizer was used with a learning rate of 0.0001, a first-moment decay rate of 0.9, a second-moment decay rate of 0.999, and an epsilon value of 10^−8^.

### 3.4. Network Architecture

**Figure 8:**
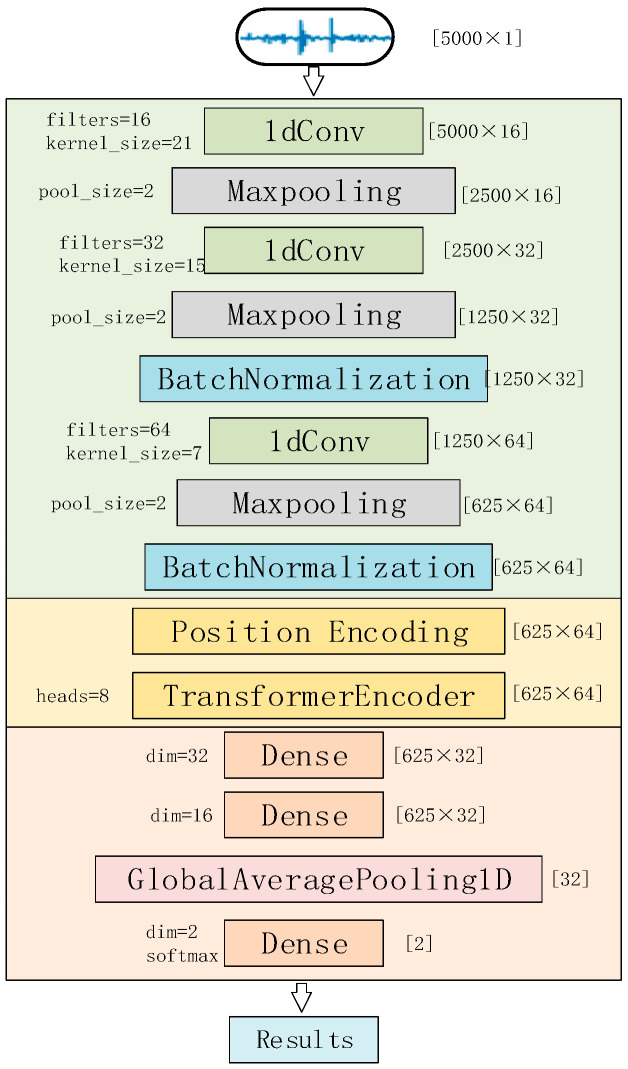
Architecture of CTENN.

The overall architecture of the Convolution and Transformer Encoder Neural Network (CTENN) is shown in Figure 8.

The network mainly consists of three modules. The preprocessed one-dimensional heart sound sequence input is sequentially passed through the one-dimensional convolution module, the Transformer encoder, and the fully connected module and then outputs the classification result.

The one-dimensional convolutional module contains three 1D-conv layers for feature extraction, three Maxpooling layers for data compression, and two BatchNormalization layers for normalization. The fully connected module contains three Dense layers for data compression and classification and a GlobalAveragePooling layer for transforming multi-dimensional features into one-dimensional features.

### 3.5. Pre-Experiment

Typically, we incorporate positional encoding after the input signal. To discuss the impact and role of positional encoding on the network, we moved the positional encoding module to the beginning of the network’s structure and conducted a set of control experiments on dataset *a*. Both models were trained for 200 epochs with a batch size of 128.

**Figure 9:**
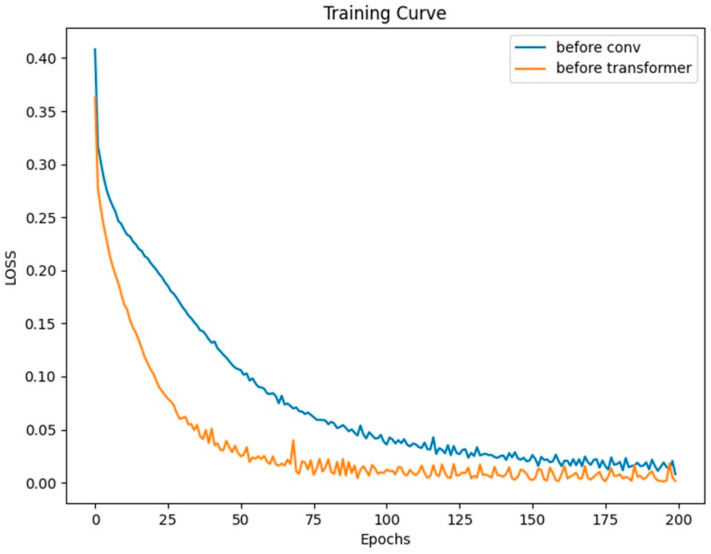
Two models’ training losses.

**Figure 10:**
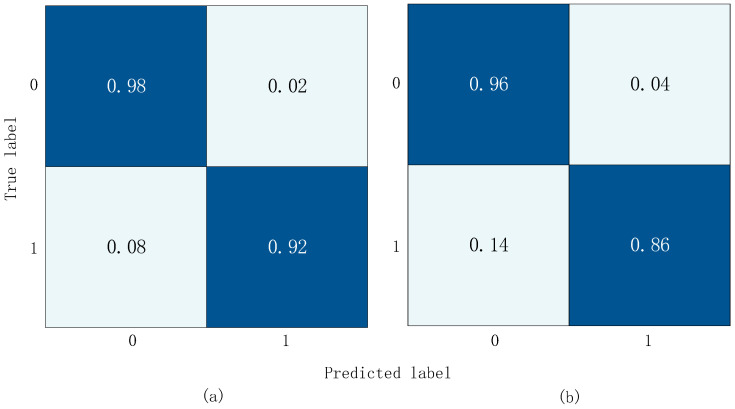
Confusion matrix results. (**a**) Position encoded before Transformer; (**b**) position encoded before Conv.

From the curves in Figure 9, it can be observed that both models eventually converge. Moreover, based on the LOSS curves and the results from testing on the validation set as shown in Figure 10, the effect of placing the positional module before the convolutional module is not as good as placing it before the Transformer layer. In this network, the convolutional layers focus more on extracting local features from the heart sound signal rather than positional information. This means that positional information has less impact on the convolutional layers. On the contrary, positional information is crucial for the Transformer module. However, after going through convolutional operations, positional information can become blurred to some extent, making it difficult for the Transformer module to accurately understand the signal’s features and positional relationships.

The output of the convolutional module is a two-dimensional signal of [625 × 64]. Here, 625 can be understood as a sequence of features with a length of 625 after the heart sound signal undergoes convolution, which corresponds to 625 tokens. The number 64 represents each token having 64 dimensions. Subsequently, the two-dimensional sequence with positional encoding is fed into the Transformer module, ensuring that the Transformer module can accurately capture the positional relationships of the signal. This is crucial for the Transformer module to learn more significant global features.

### 3.6. Experiment

Firstly, we conducted experiments on datasets *a*, *b*, and *c* separately. Dataset *a* and *c* have larger data sizes, so we set the batch size to 128. However, due to the smaller sample size of dataset *b*, the batch size was set to 32. Similarly, each dataset was trained for 200 epochs, and the training progress is shown in the graph. Ultimately, all models converged. Finally, we tested each model using the test set and obtained the final test results.

### 3.7. Results

**Figure 11:**
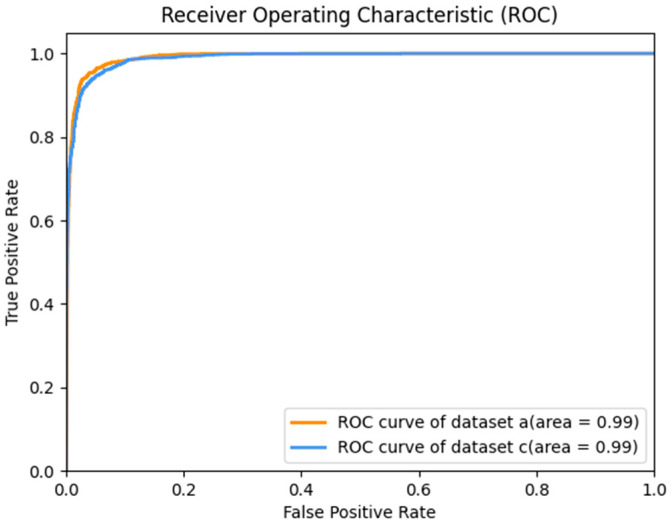
The ROC of datasets *a* and *c*.

**Figure 12:**
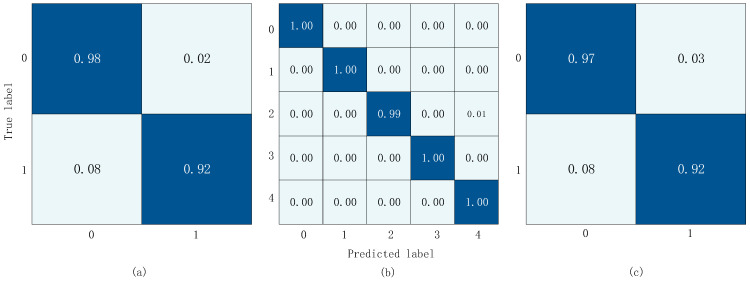
Confusion matrix results. (**a**) Confusion matrix results of dataset *a*; (**b**) confusion matrix results of dataset *b*; (**c**) confusion matrix results of dataset *c*.

The Receiver Operating Characteristic (ROC) curve of the test results of datasets *a* and *c* is shown in Figure 11. Since dataset *b* is a multi-classification dataset, the ROC curve is meaningless. If it is regarded as a one-to-many problem, that is, if the common case is regarded as a positive case and the disease as a negative case, then, Dataset *c* merges dataset *b* and other multi-classification datasets into binary classification datasets, so only the ROC curves of datasets *a* and *c* are discussed here. Also, we can see in Figure 12 that the accuracy of each category in dataset *b* is close to 1, so the AUC is very close to 1. The AUC of dataset *a* and dataset *c* is 0.99. This shows that our model is very good at distinguishing between positive and negative samples.

The confusion matrix results obtained from training and validation on three datasets are shown in Figure 12. In the diagram, the *x*-axis represents the predicted label and the y-axis represents the actual label. In Figure 12a,c, 0 represents the normal label and 1 represents the abnormal label. In Figure 12b, 0 represents the normal label, 1 represents the AS label, 2 represents the MS label, 3 represents the MR Label, and 4 represents the MVP label.

Table 2, Table 3 and Table 4 include the proposed model in this paper and some existing methods in the field of heart sound signal classification, including whether the models perform accurate segmentation of heart sound signals, feature pre-extraction methods, and some result evaluation metrics.

From Table 2, we can see that the accuracy of methods [22,23,24] is not very high, indicating that the accuracy of segmentation has a significant impact on the classification accuracy. For methods that do not perform segmentation on the heart sound signal, the pre-extracted MFCC features significantly improve the classification accuracy. However, using only convolutional neural networks to classify the extracted MFCC features is limited by the convolution operation, which mainly operates on the local receptive field and local filter of the input data. This helps to capture local features of the input data but leads to a lack of global context information in the network. For the heart sound classification task, global context information is essential for accurate classification.

The methods in [14,15] do not perform segmentation or feature extraction on the signal but use GRU blocks and Clique blocks for feature extraction. Compared with LSTM and RNN, GRU and Clique can obtain better context information and more features, so they achieve good results. In this study, we used one-dimensional convolution and Transformer methods, which have better feature extraction performance than GRU and Clique, and achieved the best accuracy. Compared with the best-performing method [26], we only had 0.16 less accuracy, but because the network we used did not perform feature extraction on the heart sound signal, it can be easily applied to various heart sound classification devices.

As dataset *b* is a multi-class dataset, some evaluation metrics cannot be calculated, so we only compare the accuracy. We can see that since the number of data entries for each label in the dataset is the same, i.e., the dataset is balanced, the recognition accuracy of each model is excellent, and the difference between the classification methods does not have a significant impact on the recognition rate, as shown in Table 3. Our model can achieve an accuracy of 99.70%, which, compared with the methods in [28,29], although only slightly lower than our accuracy by 0.2 and 0.03, they still perform feature extraction in their methods.

The data in Table 4 were obtained from dataset *c*. As it is a merged dataset, there are some differences among the datasets, which led to a slightly lower overall accuracy compared with dataset *a*. However, our proposed method still achieved an accuracy of 95.7%, which is the highest among these methods, with a high score of 94.25, indicating that our proposed method is very stable.

Regarding the data volume input into the model, we conducted a comparison, as shown in Table 5. The aim was to identify factors that could influence the recognition performance of our model, facilitating subsequent improvements.

## 4. Discussion

From the above results, the advantages and disadvantages of our model are as follows.

First, we propose a network that connects a CNN and a Transformer encoder in series for feature extraction and classification of heart sound signals. The pre-processed signal is used as the input of the network, which greatly reduces the complexity and processing time of heart sound signal classification. The direct use of the heart sound sequence detection method can be easily deployed in hardware, which lays a foundation for the subsequent practical application.

Moreover, the network can not only use a CNN to extract local features of heart sound signals but also use a Transformer encoder to extract signal context information. It makes it possible to extract multiple features which are difficult to extract using traditional methods. For complex heart sound signals, the more features extracted, the more accurate the classification will be. This is also reflected in the classification results. In the three datasets, no matter the binary classification task or the multi-classification task, the classification effect of our model is excellent.

Secondly, we discuss the position of positional encoding. Experiments show that when position coding is placed before the convolution layer, the position information of the signal will become confused after convolution. This will directly affect the convergence of the model and the effect of classification.

Then, although the accuracy of the model is high, from the results, we can find that the sensitivity of the method adopted in this paper is low. Generally speaking, the low sensitivity of the model means that the accuracy of the model is not high when identifying positive samples, that is, the false negative is large. There are two possible reasons for this: (i) data imbalance, where the difference in the number of positive and negative samples is huge; (ii) the models are too simple to capture complex relationships in the data.

As shown in Table 5, we can find that the number of samples in the dataset is relatively uneven, and the number of negative samples is nearly three times that of positive samples. The data of dataset b is relatively uniform, and the effect of dataset b is very good, so the reason for the low sensitivity of this model is very likely to be the imbalance of dataset.

Finally, our method still performs well with small amounts of data. Just like dataset b, even though there are only 1000 heart sounds, we can model and classify them accurately. This can be easily deployed in different adjuncts. Such as mobile phone remote diagnosis and treatment, users only need a heart sound collector, that is, to roughly understand whether their heart sound is normal or to judge which type of disease is to remind patients to seek medical attention as soon as possible.

## 5. Conclusions and Prospects

The CTENN proposed in this paper automatically extracts features from preprocessed heart sound sequences. Without precise heart sound signal segmentation and feature pre-extraction, it reduces the complexity of the classification. Local features of heart sound signals are extracted using multiple one-dimensional convolution layers. And, a Transformer encoder is used to extract more global features of heart sound signals. The proposed method achieves excellent classification accuracy, with accuracies of 96.4% and 95.7% on binary classification datasets and 99.7% on multi-classification datasets, respectively. Experimental results show that the proposed method has better performance and generalization ability than existing methods of the same type, demonstrating its effectiveness.

Also, there is ample room for improvement in this method. For instance, using more advanced filters such as Double-Density Discrete Wavelet Transform, could enhance the purity of the heart sound signals while ensuring the retention of more information. Furthermore, undersampling and oversampling techniques can be employed to adjust the sample quantities of different categories in the dataset, thus achieving a more balanced distribution and heightened sensitivity. Additionally, optimization of the network’s parameter count can facilitate easier deployment on hardware devices.

## Figures and Tables

**Table 1:** Number of samples in each dataset.

Dataset Name	Wav Number	After Processed	Train Number	Validation Number	Test Number
dataset *a*	3126	53,353	42,682	5335	5336
dataset *b*	1000	1668	1334	167	167
dataset *c*	4711	56,172	44,937	5617	5618

**Table 2:** Results of methods on dataset *a.*

Related Work	Accurate Segmentation	Feature Pre-Extraction	Method	Se (%)	Sp (%)	Score (%)	Accuracy (%)
Maknickas [22]	No	MFCC	CNN	80.63	87.66	84.15	84.15
Alasker [23]	Yes	AlexNet Model- Extracted	AlexNet + SVM	83.71	89.99	86.85	87.65
Noman [24]	Yes	MFCCs, Time- Frequency	Markov-Switching Model	93.70	89.90	91.80	91.20
Zhang [25]	No	Temporal Quasi- Periodic Features	LSTM	96.15	93.18	94.66	none
Deng [26]	No	MFCC	RNN+CNN	98.66	98.01	98.34	98.00
Li [14]	No	No	HSSFN, HSPFN	97.87	92.45	95.16	95.50
Xiao [15]	No	No	CNN-Clique	86.21	95.16	90.69	93.28
proposed method	No	No	CTENN	92.87	97.45	95.16	96.40

**Table 3:** Results of methods on dataset *b.*

Related Work	Accurate Segmentation	Feature Pre-Extraction	Method	Accuracy (%)
son [19]	No	MFCC + DWT	deep model	92.10
Oh [27]	No	no	1-d CNN	97.00
Mu [28]	No	MFCC	1-d CNN	99.50
Minh T [29]	No	Mel spectrogram	LSTM	99.67
proposed method	No	no	CTENN	99.70

**Table 4:** Results of methods on dataset *c.*

Related Work	Accurate Segmentation	Feature Pre-Extraction	Method	Se (%)	Sp (%)	Score (%)	Accuracy (%)
Xiang [30]	no	Mel spectrogram	Xception + transfer learning	none	none	none	94.36
Wu [31]	no	MFCC	Ensemble of CNN	91.73	87.90	89.81	none
Li [32]	no	SFTF	CNN	88.70	86.40	87.55	86.00
li’ F [33]	no	MFCC	Resnet	92.32	95.47	93.90	94.43
proposed method	no	no	CTENN	91.30	97.20	94.25	95.70

**Table 5:** Number of dataset a and c samples.

Positive/Negative	Label	Total	Train
0	noramal_a	40,758	32,591
1	abnoramal_a	12,597	10,093
0	noramal_c	42,108	33,702
1	abnoramal_c	14,066	11,237
0	normal_b	432	343
1	AS_b	256	211
2	MS_b	310	239
3	MR_b	342	276
4	MVP_b	328	265

## Data Availability

The data of CTENN are available from the author upon reasonable request. The data of 2016 PhysioNet/CinC Challenge that support the findings of this study are openly available at https://physionet.org/content/challenge-2016/1.0.0/, accessed on 2 March 2023, reference number [9]. The data that support the findings of this study are openly available at https://github.com/yaseen21khan/Classification-of-Heart-Sound-Signal-Using-Multiple-Features-, accessed on 25 March 2023, reference number [20], and http://www.peterjbentley.com/heartchallenge/index.html, accessed on 2 March 2023, reference number [21].
